# Molecular Mechanisms of Cancer-Induced Sleep Disruption

**DOI:** 10.3390/ijms20112780

**Published:** 2019-06-06

**Authors:** William H. Walker, Jeremy C. Borniger

**Affiliations:** 1 Department of Neuroscience, West Virginia University, Morgantown, WV 26506, USA; William.Walker2@hsc.wvu.edu; 2 Department of Psychiatry & Behavioral Sciences, Stanford University School of Medicine, Stanford, CA 94305, USA

**Keywords:** breast cancer, sleep, IL-6, hypocretin/orexin, leptin, EEG, autonomic nervous system

## Abstract

Sleep is essential for health. Indeed, poor sleep is consistently linked to the development of systemic disease, including depression, metabolic syndrome, and cognitive impairments. Further evidence has accumulated suggesting the role of sleep in cancer initiation and progression (primarily breast cancer). Indeed, patients with cancer and cancer survivors frequently experience poor sleep, manifesting as insomnia, circadian misalignment, hypersomnia, somnolence syndrome, hot flushes, and nightmares. These problems are associated with a reduction in the patients’ quality of life and increased mortality. Due to the heterogeneity among cancers, treatment regimens, patient populations and lifestyle factors, the etiology of cancer-induced sleep disruption is largely unknown. Here, we discuss recent advances in understanding the pathways linking cancer and the brain and how this leads to altered sleep patterns. We describe a conceptual framework where tumors disrupt normal homeostatic processes, resulting in aberrant changes in physiology and behavior that are detrimental to health. Finally, we discuss how this knowledge can be leveraged to develop novel therapeutic approaches for cancer-associated sleep disruption, with special emphasis on host-tumor interactions.

## 1. Introduction

**Figure 1:**
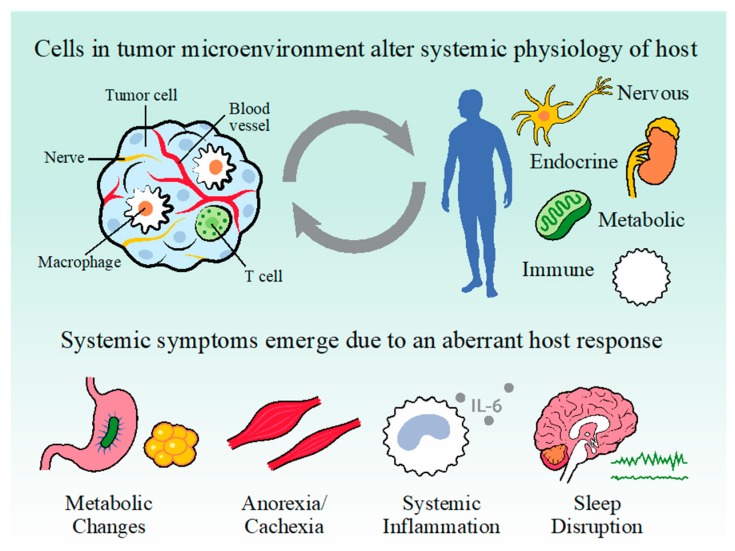
Cancer in the periphery dynamically interacts with nervous, endocrine, metabolic, and immune systems (NEMI) to elicit systemic changes in physiology and behavior. Tumor cells and those comprising its microenvironment secrete cytokines, growth factors, chemokines, and metabolites that the brain is sensitive to. This homeostatic challenge promotes aberrant neural activity, which then contributes to devastating symptoms like sleep disruption, inflammation, anorexia/cachexia, and changes in metabolism.

Tumors alter the activity of cells in their local microenvironment (e.g., T-cells, fibroblasts, macrophages) and distal organs (e.g., liver, brain) in order to evade the immune system and meet metabolic demands (Figure 1; reviewed in [1,2]). In this way, tumors present a heterogenous and dynamic physiological challenge, where collateral damage from the host response contributes to debilitating problems like fatigue, sleep and circadian disruption, impairments in energy balance, inflammation, reduced food intake, and cachexia/anorexia [3,4,5,6]. Of these, sleep disruption is among the most common, especially within breast cancer patient populations [7]. Unfortunately, poor sleep is associated with impaired patient quality of life and mortality even when controlling for multiple factors like metastatic spread, age, cortisol concentrations, estrogen receptor expression, and co-morbid depression [8,9].

It has been difficult to tease apart cause and effect in cancer-associated sleep disruption. Due to the heterogeneity among cancer types, treatment regimens, patient populations, and other lifestyle factors, the underlying mechanisms remain unclear. Indeed, a ‘chicken or the egg’ phenomenon has emerged whereby cancer seems to promote disrupted sleep, and reciprocally, poor sleep promotes tumorigenesis and cancer progression [10,11]. In this review, we provide a brief overview of sleep neurocircuitry, common sleep troubles in patients with cancer, how signals in the periphery communicate with the brain, recent mechanistic studies in animal models, and discuss further research that is necessary in treating sleep problems associated with cancer. 

## 2. Sleep Neurocircuitry

Sleep is ubiquitous across nearly all life, highlighting its ancient and important role across the phylogenetic tree. To put the following sections in context, we will give a brief overview of relevant neural circuits involved in sleep/wake control. We focus on the mammalian system, but significant work has been done in invertebrates (e.g., *C. elegans*, *D. melanogaster)*, and non-mammalian vertebrates (e.g., *D. rerio*). In mammals and some non-mammalian vertebrates, sleep can be objectively measured using electroencephalogram (EEG) and electromyogram (EMG) biopotential signals. 

During non-rapid eye movement (NREM) sleep, the firing rate of cortical neurons steadily declines compared to that observed in rapid eye movement (REM) sleep or wakefulness [12,13,14]. The EEG serves as a representation of the aggregate firing of cortical neural circuits, depending on a ‘cortico-thalamo-cortical’ loop influenced by local pacemakers and subcortical neuromodulators [15,16]. It can be split into conventional bandwidths describing cortical firing rates at different approximate frequencies, including delta (0.5–4 Hz, and containing slow waves), theta (6–9 Hz), alpha (9–12 Hz), sigma (spindle band; 12–15 Hz), beta (12–30 Hz), low (30–60 Hz) and high gamma (60–100 Hz). The synchronization of cortical firing (e.g., in the delta band) during NREM sleep depends on the precise timing of thalamocortical activity [17]. Indeed, during NREM sleep, delta waves form primary components of the EEG, with high amplitude and low frequency waves being the most prominent. In contrast, REM sleep is dominated by low amplitude theta waveforms in the EEG. REM sleep is also called ‘paradoxical sleep’ as the EEG looks similar to what one would observe during wakefulness, but the animal is deep asleep. During wakefulness, EMG activity is high and the EEG displays task-dependent spectral properties. Importantly, sleep is a homeostatic process (i.e., process S), where delta activity in the NREM EEG increases in amplitude relative to the duration of prior waking, although the mechanisms governing this process are unclear [18,19]. Sleep is also under the control of the master circadian clock (i.e., process C), ensuring that the timing of sleep coincides with environmental inputs (e.g., light, food availability). 

There are two primary subcortical brain structures that regulate arousal state stability, as well as transitions into and out of NREMS, REMS, and wakefulness. The first is the hypothalamus, which primarily serves a homeostatic function acting to adaptively regulate thermoregulation, hunger and appetite control, reproductive behavior, motivation, and sleep, among others. The second is the brainstem, where the ascending reticular activating system originates, and cholinergic signaling plays a major role in wakefulness and REM sleep control. Below, we discuss a few specific neural populations expressing neuromodulators (e.g., hypocretin/orexin) that serve to powerfully control arousal states. A full discussion of all relevant circuitry, however, is beyond the scope of this review (for more detail see: [20,21,22]).

### 2.1. Hypocretin/Orexin (HO) Neurons

The lateral hypothalamus contains numerous neural populations that receive, integrate, and fire to influence systemic physiology and behavior [23]. Among the most well studied are those that express the neuropeptides hypocretin-1 and -2 (also known as orexin-A and -B; HO). Discovered by two groups at essentially the same time [21,24], these cells serve a non-redundant role in stabilizing wakefulness. The first in vivo use of optogenetics demonstrated that these neurons are essential for transitions between sleep and wakefulness; stimulation of these neurons had an awakening effect in mice while their continued inhibition induced NREM sleep [25,26]. Further, the destruction of these neurons, absence of HO, or its receptors (primarily HcrtR2), results in the debilitating sleep disorder narcolepsy with cataplexy [27,28,29]. Recently, evidence has accumulated to support the idea that narcolepsy is an autoimmune disease, as CD8+ autoreactive T-cells have been identified in human narcoleptics [30,31]. 

HO neurons are sensitive to several signals arriving from the periphery, including cytokines, leptin, ghrelin, glucose, dietary amino acids, and changes in extracellular pH and CO_2_ [32]. Afferent inputs to these neurons were mapped using a combination of tract tracing methods, uncovering major projections from the lateral septal nucleus, bed nucleus of the stria terminalis, preoptic area, multiple hypothalamic nuclei, substantia nigra and ventral tegmental area (VTA), as well as the dorsal raphe (DR) [33]. Genetic tracing revealed cell-type specific afferents arriving from cholinergic neurons in the laterodorsal tegmentum, preoptic GABAergic neurons, as well as 5-HT+ neurons in the raphe nuclei, suggesting a major role for these neurons in functions ranging from neuroendocrine control to arousal and metabolic regulation [34,35,36]. Subsequent studies revealed that their primary arousal promoting effects are mediated though direct synaptic connections with noradrenergic neurons in the locus coeruleus (LC-NE), as HO-mediated wakefulness can be blocked via simultaneous photoinhibition of LC-NE neurons [37,38]. 

**Figure 2:**
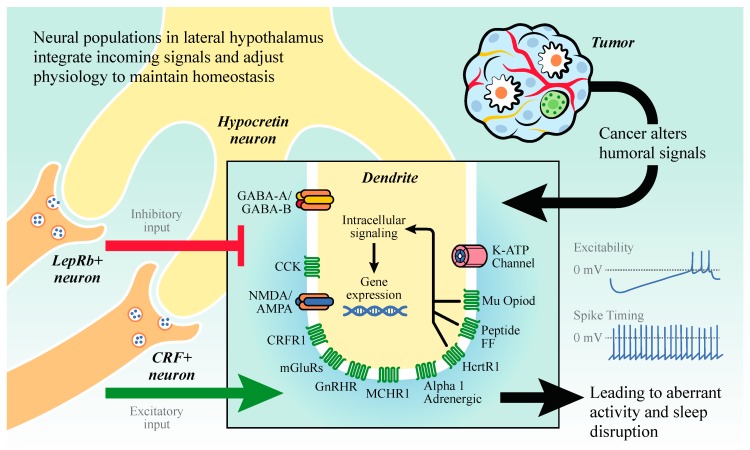
Discrete neural circuits integrate cancer-related neural and humoral signals arriving from the periphery. Depending on the timing and salience of these inputs, changes in gene expression and firing properties (e.g., spike timing) occur in an attempt to restore homeostasis. If this occurs chronically, it can influence systemic physiology and behavior resulting in debilitating symptoms like sleep and metabolic disruption.

Two key efferent outputs from HO neurons drive changes in peripheral physiology relevant to cancer. One is through engagement of the hypothalamic–pituitary–adrenal (HPA) axis to elicit secretion of glucocorticoids. Indeed, optogenetic stimulation of HO neurons rapidly promotes corticosterone secretion, elicits an aversive behavioral response, and this effect can be attenuated via leptin pre-administration [39,40]. As glucocorticoids have pleiotropic effects on the immune system [41], states of hyperarousal (e.g., anxiety, fear, panic, insomnia) can have real effects on peripheral physiology relevant to cancer. Additionally, HO neurons innervate multiple autonomic output nuclei in the brainstem, and are able to signal via the sympathetic nervous system (SNS) to alter the whole-body energy balance [42]. The disinhibition of HO neurons can promote hepatic gluconeogenesis and increase circulating glucose concentrations via the SNS [43]. Therefore, HO neurons are situated to receive signals from the periphery on energy balance and immune status, integrate these inputs, and fire to adjust arousal state and metabolic function accordingly (see Figure 2).

### 2.2. Melanin Concentrating Hormone (MCH) Neurons

Co-mingled among HO neurons are cells identified based on their expression of melanin concentrating hormone (MCH) [44]. MCH neurons are strongly active during REM sleep, somewhat during NREM sleep, and are silent during wakefulness [45,46]. This pattern is reciprocal to that of neighboring HO neurons. MCH knockout mice show REM sleep abnormalities, a reduction in NREM sleep and an increase in wakefulness [47]. MCH-containing cells are also sensitive to signals arriving from the periphery (e.g., glucose), as we discuss in subsequent sections, which give them a broader role in the regulation of energy balance and feeding behavior. We were unable to detect changes in MCH neural activity in a mouse model of non-metastatic breast cancer despite changes in sleep, however, technical limitations may have prevented us from detecting changes happening in these neurons on shorter timescales [10]. As there is evidence of inhibitory feedback between HO and MCH neurons in vitro [48], this cross-talk may serve to support appropriate coordination of sleep/wake transitions with the integration of signals of changes in systemic physiology. 

As we discuss below, cognitive (including memory) impairments are prevalent in patients with cancer, even prior to treatment initiation [49]. Kosse and Burdakov recently demonstrated that MCH neurons are critical for encoding object location memories [50]. MCH neurons increase activity (measured via GCaMP6s fluorescence) during novel object exploration. The closed-loop inhibition of these neurons during natural object exploration prevented the formation of object location memories, a process that is regulated by local inhibitory GAD65+ neurons in a GAD65→MCH circuit. As MCH neurons are sensitive to peripheral inputs (including glucose) that become deregulated in cancer, their dysfunction may contribute to sleep and memory impairments experienced by patients with cancer. 

### 2.3. VLPO GABAergic Neurons 

Sherin and colleagues identified a group of sleep-active neurons in the ventrolateral preoptic area (VLPO) that synapse onto histaminergic neurons in the tuberomammillary nucleus (TMN) [51]. These neurons contain the inhibitory neurotransmitters GABA and galanin, and innervate other components of the ascending arousal system including the locus coeruleus (LC), the raphe, periaqueductal gray, parabrachial nuclei, and the lateral hypothalamus (including HO neurons) [52]. Structurally, the VLPO is comprised of a dense core of sleep-active, galanin+ neurons that primarily project to the wake-promoting TMN, surrounded by a more diffuse population projecting to other targets like the dorsal raphe and LC [53]. Cell type specific lesion studies suggest that neurons within the core are most closely associated with NREM sleep, and those in the extended VLPO are associated with REM sleep, as destruction of these cells suppressed NREM and REM sleep by 50% or more, respectively. Although they are intermingled with other neurons that do not show arousal-state dependent changes in firing rate, VLPO ‘sleep-active’ neurons fire at about 1–2 Hz during wakefulness, 2–4 times faster during NREM sleep, even more frequently during NREM sleep following sleep deprivation, and the fastest during REM sleep [54].

Like many other populations in the hypothalamus, VLPO neurons integrate physiological signals that become deregulated in the context of cancer. For example, elevations in extracellular glucose concentrations increases cFos expression in putative ‘sleep active’ VLPO neurons, without similar changes in neighboring nuclei (e.g., LPOA, MPOA) [55]. The infusion of physiological concentrations of glucose into the VLPO promotes NREM sleep, an effect that seems to be driven by closure of potassium gated ATP channels (K_ATP_). This suggests that multiple hypothalamic nuclei (both wake and sleep-promoting) monitor changes in systemic energy balance to adjust arousal state. Logically, cancer-induced changes in metabolism, immunity, or endocrine function likely disrupt sleep via the promotion of aberrant activity within these neural populations (see Figure 1). 

### 2.4. VTA 

The ventral tegmental area (VTA) in the midbrain has only recently been linked to sleep and sleep-related behaviors. Early reports suggested that both dopaminergic (DA) and GABAergic cells within this region are maximally active during REM sleep, followed by wakefulness, and relatively silent during NREM sleep [56,57]. Whether these neurons played an active role in regulating arousal states, however, was unknown. In the last couple of years, advances in technology have allowed researchers to determine that VTA-DA neurons are indeed most strongly active during REM sleep, and activation of these neurons strongly promotes wakefulness through prominent projections to the nucleus accumbens [54]. Notably, the chemogenetic silencing of these neurons caused mice to engage in ‘sleep preparatory behavior’, involving nest building, prior to sleep [58]. Co-mingled GABAergic and glutamatergic neurons (VTA-GABA/Glut) also causally contribute to arousal state dynamics, via their projections to the nucleus accumbens and lateral hypothalamus [59,60]. 

The VTA plays a critical role in motivation and goal-directed behaviors, processes that are fundamentally coupled to arousal [61]. A component of cancer-associated fatigue is a reduced motivation to complete everyday tasks (e.g., doing laundry, working, cooking) [62,63,64]. Although a systematic investigation of this midbrain circuit in cancer is lacking, reduced dopaminergic output from the VTA could underlie both reduced arousal and motivation in cancer-associated fatigue. Additionally, although VTA neurons are not classically associated with the integration of peripheral physiological signals, recent evidence suggesting that they are able to influence the systemic immunity may prove important in developing novel therapeutics for cancer [65,66,67]. 

### 2.5. Dorsal Raphe

Initial research suggested that serotonin (5HT) neurons in the raphe nuclei promote sleep, as lesions of this area or 5HT depletion could cause an insomnia phenotype in cats and rats. Later, it was shown that this effect was driven by the effects of 5HT on thermoregulation, as the insomnia phenotype only emerged in cool, but not warm environments [68]. Now, it seems that evidence points to a wakefulness-promoting role for 5HT, as it directly excites other wake-promoting circuits and SSRIs (which increase 5HT concentrations) are generally wake-promoting. Indeed, the optogenetic activation of 5HT neurons drastically increases wakefulness at the expense of NREM sleep [69], an effect that may depend on the co-release of glutamate [70].

More recently, a role for dopaminergic signaling from the raphe has been implicated in sleep-wake regulation. Indeed, dorsal raphe dopaminergic (DRN-DA) neurons (which are distinct from those expressing 5HT) are activated by salient stimuli regardless of valence (i.e., positive, negative, neutral). Further, they are most active during wakefulness, and optogenetic stimulation of these neurons rapidly promotes wakefulness, while chemogenetic inhibition induces sleep even in the presence of salient stimuli [71]. 

The raphe nuclei are sensitive to inflammatory insults originating in the periphery (e.g., cytokine release by tumor-associated macrophages) [72,73,74]. Interleukin-1 signaling (primarily IL-1β), as we discuss below, is a powerful sleep-modulatory molecule [75,76,77,78]. It interacts with many neural systems to increase NREM sleep at the expense of REM sleep and wakefulness. IL-1 modulates the activity of key arousal-related neural populations and fast neurotransmitter actions including cholinergic, glutamatergic, monoamine, and adenosine functions. In the raphe nuclei, IL-1 inhibits 5HT signaling by enhancing GABA-induced inhibitory post-synaptic potentials. It accomplishes this by recruiting GABA_A_ receptors to the cell surface, increasing chloride (Cl^−^) uptake, and delaying the potentiation of GABA-induced Cl^−^ currents. These effects can be inhibited by the co-administration of an IL-1 receptor antagonist [1,2,3,4]. Systemic inflammation is an emerging hallmark of cancer and it is likely that changes in circulating cytokine concentrations link cancer-associated immune activation with sleep and arousal. This largely remains an open area for empirical testing.

### 2.6. LC Noradrenergic Neurons

The locus coeruleus (LC) powerfully promotes wakefulness. The arousal promoting properties of these neurons are due to norepinephrine (NE) signaling onto post-synaptic targets throughout the brain [79,80]. LC-NE neurons fire at approximately 1-3 Hz during wakefulness, have variable activity during NREM sleep, and are silent during REM sleep. Importantly, these neurons participate heavily in brain-body cross talk via the sympathetic nervous system. They receive signals on critical cues from the periphery, including afferents from the cardiovascular system and nociceptors [81,82,83,84,85]. 

Reciprocally, the LC controls autonomic function via direct projections to the spinal cord and indirect actions on autonomic nuclei including the nucleus ambiguous, dorsal motor nucleus of the vagus, the rostroventrolateral medulla, the caudal raphe, salivatory nuclei, paraventricular nucleus, the Edinger-Westphal nucleus, and the amygdala [86]. Through these projections, the LC increases sympathetic tone and suppresses parasympathetic activity. Therefore, changes in LC activity result in both the disruption of arousal states and changes in autonomic function associated with complex patterns of neural activity across the brain. 

## 3. Sleep Disruption in Patients with Cancer and Cancer Survivors

Sleep disruption is common across cancers, with the highest prevalence experienced by patients with breast cancer [7,8,62,87,88]. Indeed, patients experience approximately double the rate of sleep disturbances in comparison to the general population [89]. Treatment regimens (e.g., cytotoxic chemotherapy, radiotherapy) can exacerbate these problems, which in some cases persist for many years following treatment cessation [63,88]. Hypersomnia, insufficient sleep, along with sleep fragmentation, poor sleep efficiency, hot flashes and circadian misalignment all present (to varying degrees) throughout many types of cancer. Classifying the prevalence and etiology of these problems remains challenging and no common mechanisms have been delineated [90,91,92]. 

The most common problems related to sleep in breast cancer patients across a wide range of studies using subjective and objective measures of sleep (actigraphy, questionnaires, and polysomnography) are poor sleep efficiency (i.e., <85% time in bed spent asleep), frequent nocturnal awakenings (>15/night), extended wake after sleep onset (WASO), and daytime sleepiness [88]. Tumor physiology itself likely plays a role in the development of sleep disruption and cognitive deficits, which may explain why symptoms are sometimes evident prior to starting treatment [49]. Another complicating factor is the confusing nomenclature around related, but distinct phenomenon including fatigue, sleep disruption, and excessive daytime sleepiness (EDS), which are all frequently reported as ‘feeling tired’. Fatigue is hard to quantify as it is an ultimately subjective experience with no known biomarker, potentially causing physicians to overlook or question the importance of fatigue in disease progression and outcome [93]. For our purposes, fatigue distinguishes itself from other disorders of arousal in that it is attributed to a physiological source (i.e., not related to subjective experience or mood), and is defined as an overwhelming sense of tiredness and exhaustion that is not attenuated with subsequent sleep or rest [94]. This lack of homeostatic rebound following sleep distinguishes fatigue from generalized ‘tiredness’. 

Unfortunately, fatigue and sleep disturbances frequently occur along with other neuropsychological symptoms including depression and cognitive impairment, which may either contribute to or be the result of ongoing sleep disruption. A popular hypothesis that has gained substantial support is that cancer- or chemotherapy-induced changes in sleep are driven by inflammatory mechanisms acting at sleep/wake centers in the brain [95,96,97]. Indeed, circulating inflammatory cytokine concentrations are associated with changes in fatigue and sleep quality in breast cancer patients undergoing chemotherapy [98], and inflammatory cytokines can directly modulate sleep in humans. This provides an attractive link between cancer, chemotherapy, and sleep [99,100,101]. However, significantly more research is needed to identify the exact factors, how they interact with vigilance state circuitry in the CNS, and how this ultimately causes changes in behavior and subjective feelings of arousal. Below, we provide an overview of potential mechanisms underlying cancer-associated sleep disruption, primarily focusing on humoral signals from the immune system and those relaying changes in energy balance to the brain.

## 4. Immune Pathways Deregulated by Cancer that Influence Sleep

The tumor microenvironment, consisting of the surrounding blood and lymphatic vessels, immune cells, fibroblasts, and extracellular matrix, performs an integral role in the development of solid tumors [102]. Multiple cellular processes are required for the emergence of neoplastic tissue and the progression to malignancies; namely, limitless replication potential, adequate growth signals, insensitivity to growth-inhibitory signals, evasion of apoptosis, sustained angiogenesis, and ultimately tissue invasion and metastasis formation [2]. Notably, inflammation can affect the majority of these processes [103]. Virtually all tumors have some type of innate and adaptive immune cell infiltration. This was originally thought of as a productive immune response to elicit anti-tumor effects; however, more recent studies have demonstrated that the tumor associated immune response can instead enhance tumorigenesis and progression [2,104,105]. Cancer cells can secrete leukocyte attracting chemokines, such as C-C Motif Chemokine Ligand 2 (CCL2), CCL4, CCL5, CCL7, CCL8, and CCL20 leading to an infiltration of tumor associated macrophages, neutrophils, T cells, and dendritic cells [105,106,107]. In turn, these leukocytes secrete growth factors that promote proliferation (e.g., hepatocyte growth factor (HGF), epidermal growth factor (EGF), insulin-like growth factor (IGF), fibroblast growth factor (FGF), platelet-derived growth factor (PDGF), and transforming growth factor beta (TGF-β)), pro-angiogenic factors that increase nutrient supply (vascular endothelial growth factor (VEGF) and basic fibroblast growth factor (bFGF), anti-apoptotic factors that prevent cell death (nuclear factor kappa-light-chain-enhancer of activated B cells (NF-κB)), enzymes that break down the extracellular environment to enhance invasiveness and promote metastases (matrix metalloproteinases; MMPs and cytokines that work to enhance all of the above include interleukin-1 (IL-1), interleukin-2 (IL-2) interleukin-6 (IL-6), tumor necrosis factor alpha (TNF-α), interleukin-4 (IL-4), interleukin-8 (IL-8), interleukin-10 (IL-10), and TGF-β [2,103,104,105,106,107,108,109,110,111].

Tumor secreted cytokines and growth factors are not limited to the tumor microenvironment. Once released, cytokines and growth factors can circulate throughout the body and propagate to the brain via two main routes, humoral and neural [112,113,114]. Within humoral signaling there are multiple pathways by which peripheral cytokines can be transduced into the brain. Cytokines can enter the CNS through simple diffusion at circumventricular organs, which lack a fully functional blood brain barrier (BBB), they can bind cytokine transporters at the BBB and be transported into the brain, and they can bind cytokine receptors on endothelial cells that in turn release IL-1 and prostaglandins within the brain parenchyma. The neural route consists primarily of signaling from vagal afferents arising from the thorax and abdomen. These nerves express cytokine receptors that when activated result in a neural signal to the brain. This neural signal can be propagated or transduced back into an immune signal within the CNS. Once in the brain (via humoral and/or neural route), these cytokines activate microglia, which propagate this signal leading to alterations in behavior and sleep. Further, microglia can induce neurotoxic reactive astrocytes, which further amplify and propagate the inflammatory signal to influence neural survival, axon conductance and myelination, stem cell differentiation, and behavior [115,116,117]. 

## 5. Interleukin-1

IL-1β, IL-6, TNF-α, IL-4, IL-10, and TGF-β are among the most well studied cytokines known to effect cancer initiation/progression and sleep [72,103,118,119]. IL-1β can be produced directly by tumors or by tumor associated leukocytes [120]. High production of IL-1β by tumors is generally associated with poor prognoses [121,122]. Within the tumor microenvironment, IL-1β acts as a pleiotropic cytokine, increasing tumor growth and invasiveness via induction of MMPs, VEGF, IL-8, IL-6, TNFα, and TGFβ [120]. primarily, through NF-κB signaling [103] Indeed, in a mouse model of melanoma, IL-1β signaling was demonstrated to be necessary for in vivo angiogenesis and invasiveness [123]. As previously discussed, IL-1β is not restricted to the tumor microenvironment. It can signal to the brain via passive diffusion at circumventricular organs, binding to IL-1R1 on endothelial cells at the BBB, or by binding to IL-1Rs expressed on vagal afferents [114,124,125]. Once in the brain IL-1β can act on a multitude of sites to affect behavior and sleep. 

A central or systemic injection of IL-1β enhances both delta power (~0.5–4 Hz oscillations) during NREM sleep and the duration of NREM sleep (i.e., it acts as a somnogen). Inhibition of IL-1β via via administration of neutralizing antibodies or an IL-1β receptor antagonist reduced spontaneous NREM sleep [126,127]. However, IL-1β

β’s effect on REM sleep seems to be time of day and dose dependent. Low levels of IL-1β have no effect on the duration of REM sleep. However, high doses of IL-1β inhibits REM sleep [72]; further supporting IL-1β’s role as sleep regulatory substance. IL-1β concentrations within the brain follow a diurnal pattern, peaking when NREM sleep duration is greatest. Further, in response to sleep deprivation, IL-1β expression within the brain is increased [128]. IL-1β can act on multiple sleep nuclei. For example, microinjection of IL-1β into the dorsal raphe or locus coeruleus inhibits neural activity and enhances NREM sleep [77,129]. Further, microinjections of IL-1β reduces the activity of wake-promoting neurons in the basal forebrain and increases the activity of sleep promoting neurons in the preoptic area [130]. IL-1β can further influence a variety of other molecules and neurotransmitters that influence sleep (e.g., NF-κB, cyclooxygenase-2, nitric oxide (NO), adenosine, prostaglandins, and GABA). For example, IL-1β increases NO production and administration of L-NAME, an inhibitor of nitric oxide synthesis, reduces IL-1β induced NREM sleep [131]. Together, these data demonstrate that IL-1β acts as a NREM sleep promoting molecule, which is under circadian and homeostatic control.

## 6. Interleukin-6

Interleukin-6 is an inflammatory and pleiotropic cytokine, with tumor stimulating and inhibitory effects [132,133]. IL-6 is commonly produced by a variety of cancer types including breast, lung, liver, and prostate cancer and elevated serum IL-6 is generally correlated with poor outcomes in cancer patients [134,135,136,137]. Within the tumor microenvironment, IL-6 is secreted by tumor associated macrophages (TAMs), T-cells, fibroblasts, and malignant cells (i.e., cancer cells). Specifically, TAMs secrete IL-6 to aid in tumor promotion, whereas, during tumor progression T-cells become the primary source of IL-6 [103,138,139]. It is important to note that IL-6 signaling can occur via classical or trans-signaling pathways [140]. During classical singling, IL-6 binds to the membrane bound IL-6 receptor which then binds to the glycoprotein 130 (gp130 subunit) and allows for signal transduction. Classical signaling occurs in the liver and some leukocytes express membrane bound IL-6. However, during trans signaling, the major signaling pathway used within the tumor microenvironment and CNS, IL-6 binds in solution to a soluble Il-6 receptor (sIL-6R) which is secreted by cells. This IL-6/sIL-6R complex can bind to gp130 expressed by most cells types and can induce IL-6-mediated signaling in those cells. IL-6 secretion is induced by a multitude of factors, including lipopolysaccharides (LPS), prostaglandins (PGE-2), hypoxia, oxidative stress, VEGF, TNFα, and IL-1β [132]. Once released, IL-6 aids in tumor promotion and progression by activating major proliferative pathways (STAT3, MAPK, and PI-3K), inhibiting many pro-apoptotic mediators (p53 and forkhead box (FOX) proteins) via AKT signaling, and inducing the activation of anti-apoptotic genes (Bcl-2, Bcl-xL, and Mcl-1) via STAT3. Indeed, studies have demonstrated that IL-6 and its downstream signaling transcription factor, STAT3, are essential for the formation and progression of liver cancer, lung cancer, breast cancer, and leukemia [141,142,143,144]. Furthermore, IL-6 production by cancer cells has detrimental effects such as resistance to chemotherapeutics and eventual tumor relapse [145,146]. Similar to IL-1β, IL-6 is not restricted to the tumor microenvironment. IL-6 signaling to the brain is thought to occur primarily through humoral signaling as evidence of IL-6 signaling via the vagus nerve is scarce [147,148].

Interleukin-6′s role in sleep is not yet thoroughly understood. In humans, IL-6 plasma concentrations follow diurnal rhythms. IL-6 is low during wakefulness and peaks during sleep [72,149]. Similar to IL-1β, sleep deprivation increases the amount of circulating IL-6 [150,151]. Subcutaneous injections of IL-6 in humans increases slow wave sleep (defined as the total amount of stage III and IV sleep) and reduces REM sleep [72,152]. However, animal models investigating the effects of IL-6 on sleep have produced conflicting results. Indeed, ICV injection of human recombinant IL-6 into rabbits demonstrated a pyrogenic but not somnogenic effect [153]. However, ICV injection of rat recombinant IL-6 into rats temporarily enhances NREM sleep followed by a subsequent reduction of NREM sleep [154]. Furthermore, blocking IL-6 signaling via neutralizing antibodies had no apparent effect on natural sleep [Interleukin-6 alters sleep of rats.]. Notably, the relationship between sleep and IL-6 is not unidirectional. In humans sleep enhances IL-6 trans-signaling with little to no effect on classical/membrane bound IL-6 signaling. Indeed, sleep greatly enhanced the concentrations of sIL-6R, exceeding wake levels of sIL-6R by 70% at the termination of sleep [155]. This likely reflects sleep’s support of immune defenses as there is an increasing amount of evidence demonstrating a positive role for sleep in immunity [156,157]. Together, the data from human an animal models suggest that IL-6 influences sleep in a time-of-day and dose-dependent manner. 

## 7. Tumor Necrosis Factor 

Tumor necrosis factor is a proinflammatory cytokine with pro- and anti-tumor effects. In fact, TNF was first isolated in 1975 by Carswell and colleagues while studying the hemorrhagic necrosis of tumors [158]. The authors demonstrated that TNF-positive serum is just as effective as endotoxin in promoting necrosis in a variety of tumors. The authors postulated that macrophage derived TNF mediated the anti-tumor effects. Additional studies using high doses of TNF replicated TNF’s anti-tumor effects. Indeed, exogenous administration of human recombinant TNF to mice induced necrosis in xenografted and syngeneic tumors [159,160,161]. However, to be effective TNF had to be injected repeatedly and locally. Upon further investigation, administration of exogenous recombinant TNF of the same species (i.e., recombinant mouse TNF to mice) produced severe toxicity [162]. It was initially believed that TNF mediated anti-tumor effects via direct cytotoxic or cytostatic actions on malignant cells. However, this was later demonstrated to be incorrect as TNF can promote resistance and resilience in cytotoxic conditions [163]. Additional evidence supporting TNF’s pro-tumor role came from studying TNF-KO mice. Moore and colleagues demonstrated that mice lacking TNF treated with a skin carcinogen actually developed fewer rather than more tumors [164]. Substantial evidence has accumulated demonstrating TNF’s pro-tumor effects in animal models [161]. Within the tumor microenvironment, TNF is produced by tumor associated macrophages and is constitutively produced in cancer cells [165,166]. Through activation of NF-κB, TNF can induce the expression of a variety of pro-tumor genes including MMPs, COX2, and VEGF. Further, activation of NF-κB promotes cell survival through its anti-apoptotic actions [167]. More recent evidence suggest that TNF can bind to TNF receptor 2 (TNFR2) expressed predominately on regulatory T-cells (Tregs) to suppress anti-tumor immunity [168,169]. As expected, TNF is not restricted to the tumor microenvironment and can signal to the brain via humoral routes. Indeed, studies have demonstrated that TNF can be transported across the BBB, where the inflammatory signal is further propagated across the brain parenchyma [170,171]. 

Tumor necrosis factor has a well demonstrated somnogenic effect. In humans, plasma TNF concentrations correlate with EEG slow wave activity [172]. Additionally, studies in rats have demonstrated diurnal rhythms in TNF concentrations within the hypothalamus, with peak concentrations observed during sleep [173,174]. TNF’s ability to promote NREM sleep was first described by Shoham and colleagues [175]. They observed that the administration of human recombinant TNF to rabbits via IV or ICV injection enhanced slow wave sleep with concurrent reductions in REM sleep and biphasic fevers. Additional studies suggest that TNF can also enhance slow wave sleep in rats and mice [176,177]. Increases in NREM sleep following TNF administration is generally accompanied by concurrent reductions in REM sleep; however, low dose administration of TNF to mice does not affect REM sleep. Similar to IL-1, TNF can act on multiple sites within the brain to enhance sleep. For example, microinjection of TNF into the preoptic area in rats increases NREM sleep [177]. Further, the administration of sTNFR fragment into the preoptic area reduces NREM sleep. TNF can also act on wakefulness promoting regions; specifically, elevations in TNF concentrations decreases the mRNA half-life and enhances protein ubiquitination and subsequent degradation of wake-stabilizing hypocretin-1 and hypocretin-2 (discussed above) [178]. Additionally, microinjections of human recombinant TNF into the locus coeruleus of rats enhanced sleep; this effect was blocked by pre-treatment with polyclonal antibodies against TNF [179]. Furthermore, infusions of TNF into the subarachnoid space near the rat basal forebrain increased slow wave sleep and reduced REM sleep [180]. Similar to IL-1, TNF can have indirect effects on sleep through the activation of downstream molecules such as COX or NO [181]. Co-infusion of TNF and a non-selective cyclooxygenase (COX) inhibitor or pretreatment with a COX-2-specific inhibitor into the subarachnoid space near the rat basal forebrain blocked TNF-mediated increases in slow wave sleep. Together, these data demonstrate that TNF is a somnogenic cytokine that increases NREM sleep at the expense of REM sleep and wakefulness.

## 8. Transforming Growth Factor Beta, Interleukin-4, and Interleukin-10

TGFβ, IL-4, and IL-10 are anti-inflammatory pleiotropic signaling molecules that are involved in critical functions during tumor promotion and progression. The role of these signaling molecules as tumor promoting or tumor suppressing are still being debated, as these signaling proteins display differential effects during the early and late stages of tumor development. For example TGFβ early in tumor development is associated with a better prognosis due to its effects on cell cycle arrest and apoptosis [182]. However, later-stage tumors with high TGFβ concentrations are associated with increased aggressiveness and more metastasis [183]. TGFβ is produced by malignant cells and macrophages in order to increase angiogenesis via the upregulation of VEGF and bFGF, suppress the immune system via multiple steps (driving T-helper cells and macrophage polarization towards a Th2 and M2 phenotype, increasing activation of T-reg cells, and reducing cytotoxic activity of CD8+ T-lymphocytes and natural killer cells), and promote metastases via the activation of signaling proteins (Smads) necessary for epithelial to mesenchymal transitions [118].

Similar to TGFβ, IL-10 and IL-4 have dynamic effects on tumor promotion and progression. For example, elevated concentrations of systemic IL-10 are associated with a poor prognosis, but paradoxically, high levels of tumor IL-10 are associated with a better prognosis [184]. Additionally, studies examining IL-4 concentrations in the blood of breast cancer patients before starting treatment demonstrate a correlation between IL-4 and subsequent mortality [185]. Other studies examining IL-4′s role in prostate cancer suggest that serum IL-4 concentrations are elevated in patients with benign prostatic disease [186], and IL-10 and IL-4′s pro tumor effects likely reflect their immunosuppressive properties. However, these same properties can result in paradoxical anti-tumor effects as well. Thus, the actions of IL-10 and IL-4 on tumors are varied and are still a subject of ongoing investigation (see [187,188,189]). Few studies have examined the ability of IL-10 and TGFβ to be transported across the blood brain barrier (BBB) demonstrate no active transport across a normal intact mouse BBB [190]. Additionally, to our knowledge no study has examined the ability of IL-4 to be transported across the blood brain barrier [191]. Thus, any peripheral to brain signaling likely occurs at circumventricular organs. 

Contrary to IL-1 and TNF, IL-4, TGFβ, and IL-10 reduce sleep [181]. Indeed, ICV administration of IL-10 or IL-4 to rabbits during the light phase (rest phase) inhibited NREM sleep [192,193]. High doses of IL-10 (250ng) or IL-4 (250ng) administered to rabbits during the light phase inhibited NREM sleep and significantly decreased REM sleep. However, the administration of IL-10 or IL-4 during the dark phase had no effect on sleep. Similar studies examining the effects of IL-10 on sleep have replicated these finding in rats [194]. Further, studies examining TGF-β’s role in sleep suggest it has similar effects on sleep. ICV administration of TGF-β to rabbits during the light phase reduced NREM sleep but had no effect on REM sleep. Despite this, administration of TGF-β during the dark phase had no effect on sleep [195]. The mechanism by which IL-4, TGFβ, and IL-10 reduce sleep has not been elucidated. However, previous studies have postulated that these anti-inflammatory cytokines reduce sleep by inhibiting the production of IL-1 and TNF, powerful sleep-promoting components of the immune system. Together, these studies demonstrate that IL-4, TGFβ, and IL-10 are anti-somnogenic cytokines and that their sleep inhibitory properties depend on dose and time of day. 

## 9. Cancer, Energy Balance, and Sleep

While cytokines secreted by the tumor or tumor microenvironment are a rather obvious mechanism by which peripheral tumors can alter sleep, they are not the only mechanism. Tumors can also affect sleep through alterations in metabolism and subsequent energy balance. For example, recent studies have demonstrated that tumors can directly secrete ghrelin to aid in metastasis and cell proliferation [196]. Ghrelin is a peptide hormone typically produced in the stomach and brain to induce food intake and stimulate growth hormone secretion. Ghrelin is produced in two forms: acyl-ghrelin, the “active form” that serves as the endogenous ligand for the growth hormone secretagogue receptor (GHSR), and des-acyl ghrelin, the inactive form that does not activate the GHSR receptor and does not induce GH release from the pituitary [197]. It is important to point out the “inactive” form of ghrelin is a misnomer; des-acyl ghrelin has known signaling effects [198,199,200]; however the receptor that des-acyl ghrelin binds to induce downstream signaling is currently unknown. Ghrelin and its receptor GHSR are expressed in a multitude of cancers including breast, ovarian, prostate, pancreatic, oral, gastric, and colorectal cancer [196]. The effect of ghrelin and des-acyl ghrelin are varied and cancer specific. For example in human prostate cancer cell lines, ghrelin and des-acyl ghrelin inhibited cell proliferation in the DU-145 cell line but had no effect on LNCaP cells [198]. However, in other prostate, breast, and endometrium cell lines, ghrelin stimulates cell growth [201]. Additionally, the relationship between ghrelin expression and outcome in cancer patients is complex and still being elucidated. In breast cancer patients, ghrelin has been associated with favorable outcomes in recurrence and survival [202]. Whereas, in renal cell carcinoma ghrelin is associated with poor outcomes and survival [196]. Ghrelin actively crosses the blood brain barrier in humans and mice [203]. However, notably, des-acyl ghrelin crosses the BBB to a much greater extent in mice. In contrast, in humans, des-acyl and acyl ghrelin cross the BBB at equivalent rates. Similar to the effects on tumor growth and metastases, the effects of ghrelin on sleep are complex and at times contradictory. Intravenous ghrelin injections in rats and mice increase NREM sleep [204,205,206]. However, ICV injections in ad libitum-fed and fasted rats reduces NREM sleep [207]. Additionally, human data demonstrate further contradictions with elevated ghrelin concentration associated with short sleep durations; whereas the administration of ghrelin to humans increase NREM sleep [208,209]. Further additional studies demonstrate increased ghrelin concentrations in humans that have been sleep deprived [210]. The mechanisms by which ghrelin can inhibit sleep or promote sleep have not explicitly been tested. However, ghrelin can act on hypocretin neurons to increase their activity and this may explain the inhibition of sleep [211]. Additionally, as previously discussed, the systemic injection of ghrelin in mice increased sleep; however, this effect was abolished in mice lacking functional GHRH receptors, suggest ghrelin may be acting via GHRH receptors to promote sleep [206]. Together, these studies demonstrate a highly complex and at times contradictory effect of ghrelin on sleep. This complexity and contradictions are likely due to different routes of administration, the varying forms of ghrelin, and the multiple endogenous receptors of ghrelin and des-acyl ghrelin can bind.

Leptin is an additional metabolic hormone with direct effects on cancer and sleep. Leptin’s role in non-diseased animals is to function as a satiety signal and increases energy expenditure; thus, opposing ghrelin’s actions [212]. Leptin is produced via adipocytes and can signal via its receptor Ob-R. In general leptin is considered to be beneficial for tumor promotion and progression due to its shared signaling pathway with IL-6 (see above) [213]. Leptin’s receptor Ob-R is an IL-6 family receptor; thus, binding of leptin to its receptor induces the similar signaling cascade as IL-6 signaling [214]. Additionally, leptin can directly increase the production of IL-6 and TNF-α [215]. Leptin and/or its receptor have been confirmed in breast, colorectal, prostate, pancreatic, ovarian, and lung cancer [213]. Typically, leptin is associated with increased cell proliferation in cancer. However, there are studies demonstrating decreased cell proliferation in pancreatic cancer [216]. Additionally, high serum leptin levels have been associated with increased risk of breast and colorectal cancer [217,218,219]. Leptin crosses the blood brain barrier via a saturable system and may interact directly with sleep nuclei [220]. Similar to ghrelin, the effects of leptin on sleep are unclear and still under investigation. In humans leptin levels demonstrate a diurnal rhythm peaking during sleep [221] and short sleep duration is associate with reduced leptin levels [209,222,223]. Intraperitoneal administration of leptin to rats increased delta power and slow-wave sleep with concurrent reductions in REM sleep [224]. However, Laposky and colleagues examined sleep in leptin receptor deficient mice and demonstrate increased overall sleep time, increased sleep fragmentation, and alterations in delta power [225]. The mechanism by which leptin alters sleep/wake states has not been thoroughly investigated. However, leptin excites hypothalamic neurons expressing the long-form leptin receptor (LepRb), which synapse directly onto inter-mingled hypocretin/orexin neurons [39]. Significant work is still needed to understand leptin’s role in sleep and its underlying circuitry.

Other less defined mechanisms by which cancer may affect sleep include changes in glucose concentrations in the blood, amino acids concentrations in the blood, and pH levels. The metabolic requirement for cancer cells is immense; thus, cancer cells require high glucose levels and increase the demand for amino acids in order to maintain consistent proliferation. Indeed, tumors consume extreme amounts of glucose relative to healthy tissues and require exogenous and/or de novo supply of amino acids [226,227]. Intriguingly, amino acid content and blood glucose levels increase in the serum to meet the energetic demands of the tumor [228,229,230]. Additionally, acidosis (an overproduction of acid) is an hallmark of tumors to increase invasiveness, drug resistance, and proliferation [231,232]. This is thought to occur due to the high rate of glycolysis and reduced functional vasculature within the tumor. Notably, alterations in glucose, amino acids, and pH can affect sleep nuclei within the brain. Elevations in glucose inhibit hypocretin neurons via tandom-pore potassium channels [233]. Conversely, hypoglycemia increases hypocretin neuron activity [234]. Additionally, glucose can act to increase the activity of MCH neurons in the lateral hypothalamus [235]. Amino acids stimulate the activity of hypocretin neurons; indeed Karnani and colleagues demonstrated increased cFos expression in hypocretin neurons following peripheral and central administration of physiological mixtures of amino acids [236]. Further, hypocretin neurons are sensitive to changes in pH; specifically, reductions in pH increase the activity of hypocretin neurons [237]. Alterations in glucose concentrations, amino acid dynamics, and pH have not been examined with respect to cancer induced sleep alterations, and offer an exciting avenue for future research.

## 10. Preclinical Research

Despite the prevalence and severity of sleep problems in patients with cancer and cancer survivors (see prior sections), few mechanistic studies aimed at understanding this phenomenon have been conducted. Below, we discuss several examples linking cancer-induced changes in physiology to arousal circuitry in the brain. Focusing on the lateral hypothalamus, hypocretin/orexin (HO) neurons have been linked to the development of sleep and metabolic abnormalities in a mouse model of non-metastatic breast cancer [10]. Using female Balb/C mice and syngeneic mammary tumor cells (67NR, 4T1, 4T07), the authors demonstrated that peripheral tumor growth promotes systemic inflammation, largely driven by interleukin-6 (IL-6). Tumor-bearing mice exhibited phenotypes consistent with classical IL-6 signaling (hepatic), including pSTAT3 induction, *socs3*, *il1r1, il6ra,* and *ccl2* gene expression changes. This was accompanied by drastic changes in gluconeogenesis/glycolysis pathway gene expression, hyperglycemia/insulinemia, reduced locomotor activity, sleep fragmentation, and altered satiety hormone (leptin/ghrelin) signaling. 

When the brains of these mice were examined, HO neurons in the LH, which are sensitive to glucose, leptin, and ghrelin, were found to be aberrantly active. As we discussed above, cancer and cancer-related systemic inflammation is thought to drive sleep disruption and fatigue [95,97], however this had not been formally tested in a preclinical model. To test whether IL-6 was promoting changes in sleep, the researchers administered anti-IL-6 monoclonal antibodies (mAbs) or the IgG isotype control to tumor- and non-tumor bearing mice. This successfully attenuated measures of inflammation (reduced pSTAT3, *socs3*, *il1r1* expression), but was unable to rescue tumor-induced changes in sleep or glucose processing. 

However, when mice were administered a dual hypocretin receptor antagonist (Almorexant), both measures of peripheral metabolic disruption and sleep fragmentation were attenuated. This was accompanied by increased NREM spectral power in the delta band, indicative of deep, restorative sleep. If HO neurons are signaling to the periphery to influence glucose metabolism, how is that signal propagated from the brain? A likely pathway is through the sympathetic nervous system (SNS), as HO neurons send projections to diverse autonomic output nuclei in order to influence systemic physiology [42,43]. Indeed, when peripheral sympathetic nerve terminals were ablated using intraperitoneal injections of the neurotoxin 6-hydroxydopamine (6-OHDA), tumor-bearing mice no longer showed hyperglycemia, or the aberrant expression of genes involved in gluconeogenesis and glycolysis. These data demonstrate a bidirectional communication pathway between tumors in the periphery and the brain, with signals being relayed by endocrine, metabolic, and sympathetic pathways. Additionally, these data suggest that dual hypocretin receptor antagonists (e.g., Suvorexant; Belsomra) need to be assessed as potentially novel therapies for sleep and metabolic disruption in cancer. 

This study built upon prior work indicating that lung adenocarcinoma itself is able to distally alter hepatic circadian gene expression [238]. Masri and colleagues demonstrated that lung tumors similarly promote hepatic IL-6 signaling, leading to aberrant rhythms in gluconeogenesis/glycolysis gene expression in the liver. However, no evidence was presented indicating that tumors deregulate homeostatic signaling in the brain, or any specific action on discrete neural populations (such as HO). 

Recently, HO neurons have been linked to sleep fragmentation-induced cardiovascular disease [239]. McAlpine and colleagues demonstrated that chronically fragmented sleep drastically reduces the number of lateral hypothalamic HO neurons, a phenotype associated with atherosclerosis development. To delve into the mechanism linking the brain to changes in peripheral vascular physiology, they examined hematopoietic cell populations in the bone marrow. Here, they discovered a subset of pre-neutrophils that express hypocretin receptor 1 (Hcrt-R1). Importantly, these cells secrete the critical molecule colony stimulating factor 1 (CSF1), which promotes the egress of myeloid cells from the bone marrow into circulation. Sleep-disruption induced impairments in these functions (via Hcrt-R1) resulted in downstream immune dysregulation and the development of atherosclerosis. Whether a similar mechanism could explain the association of poor sleep with cancer development [8,240] remains to be determined. Importantly, this experiment directly linked arousal circuitry with hematopoiesis and systemic immunity via Hcrt-R1. 

Inflammatory signaling likely lies at the nexus of brain-tumor cross-talk, with effects relevant to sleep. Additionally, sleep apnea, a disease characterized by chronic sleep fragmentation and systemic inflammation, has been continuously linked to cancer development [241,242]. For example, chronic sleep disruption accelerates tumor growth and progression in multiple mouse models [11]. Hakim and colleagues examined interactions between sleep, immunity, and cancer using multiple syngeneic cancer models. Mice undergoing the sleep disruption protocol had higher numbers of tumor associated macrophages (TAMs) and engagement of TLR4 signaling pathways, suggesting an inflammatory mechanism. They tested whether inflammatory signaling is necessary for this effect using TRIF and MyD88 knockout mice, where sleep fragmentation-induced cancer growth was blunted, but still occurred. In TLR4 knockout mice however, the effect of sleep fragmentation on tumor progression was completely abolished. Further studies are needed to examine the reciprocal pathway, that is, to determine how the tumor itself influences sleep through these inflammatory mechanisms.

Stress circuits are also play a key role in energy mobilization and arousal. Recent research has provided substantial evidence on how psychological or metabolic stress influence cancer growth. For example, Thaker, Sood & colleagues demonstrated that psychosocial stress enhances tumor progression in several animal models through promotion of glucocorticoid and andrenergic signaling [243,244,245]. In vitro, several ovarian cancer cell lines (EG, SKOV3, 222, and HeyA8) became more invasive upon exposure to norepinephrine alone or in combination with glucocorticoids. This effect was driven (in part) via the induction of MMPs, which serve as essential regulators of angiogenesis and tissue remodeling. Inhibition of adrenergic signaling or MMP action was able to prevent the observed increase in invasiveness. When tumor phenotypes were examined in vivo, behavioral stress (restraint) enhanced tissue catecholamines, angiogenesis, tumor mass, and invasiveness (orthotopic syngeneic ovarian cancer model). Again, these effects were dependent on adrenergic signaling (via the β2-adrenergic receptor). Downstream signaling at this receptor engaged cAMP-protein kinase A (PKA) pathways, resulting in the transcription of genes integral in angiogenesis and tissue remodeling (VEGF family and MMPs). As psychological stress predictably interacts with arousal circuitry (resulting in anxiety and insomnia), therapeutic approaches (pharmacological and/or behavioral) to reduce stress and improve sleep could significantly boost the effectiveness of traditional cancer therapies.

Independent of psychological factors, metabolic stress induced by cancer-induced changes in energy balance can promote aberrant glucocorticoid signaling which suppresses anti-tumor immunity. Fearon and colleagues demonstrated that inflammation (IL-6) alters ketogenesis pathways in the liver, leading to glucocorticoid secretion, impaired anti-tumor immunity, and failure of immunotherapy (anti-PD-1/anti-PD-L1) [246]. Obradović and colleagues further demonstrated that breast cancer thrives on stress, as glucocorticoids promote tumor cell heterogeneity and metastatic seeding [247]. Importantly, this suggests that caution must be taken when using glucocorticoid-based anti-inflammatory drugs. 

Disrupted glucocorticoid secretion pattern is consistently observed in multiple cancer types, and can be used to predict subsequent mortality [4,6]. As glucocorticoid action is controlled by interactions between central and peripheral circadian clocks (in the suprachiasmatic nucleus, pineal and adrenal glands), circadian and/or sleep-targeted therapies could greatly aid in anti-cancer immunity and promote the success of cancer immunotherapy [248,249]. Additionally, stress- and sleep disruption induced adrenergic signals from the sympathetic nervous system (which are predominantly pro-tumorigenic) are also under circadian control, an aspect that could be leveraged to improve treatment effectiveness and limit side effects. 

As we alluded to earlier, the ventral tegmental area is involved in the regulation of wakefulness, motivation, and reward. It has also recently been implicated in tumor growth and progression. This connection was probed with designer receptors exclusively activated by designer drugs (DREADDs; chemogenetics). Adeno-associated viruses (AAVs) carrying cre-dependent excitatory DREADD transgenes (AAV-DJ-EF1a-DIO-hM3Dq-mCherry) were infused into the ventral tegmental area of tyrosine hydroxylase::Cre (TH::Cre) mice, allowing for specific transgene expression only in dopaminergic VTA neurons. Using this approach, Rolls and colleagues demonstrated that chemogenetic activation of VTA-DA neurons enhanced both innate and adaptive immunity 24 h post-CNO administration [65]. When this manipulation was repeated throughout the course of tumor growth (LLC or B16 syngeneic cells), VTA-DA ‘activated’ mice had smaller tumors and altered immune systems characterized by reductions in tumor associated myeloid derived suppressor cells (MDSCs) [66]. VTA activation promoted sympathetic (norepinephrine-mediated) inhibition of MDSCs in the bone marrow, which normally act to suppress anti-tumor immunity. Finally, adoptive transfer of ‘VTA-activated’ MDSCs into naïve mice recapitulated the anti-tumor effect of DREADD activation. These exciting findings need to be more thoroughly investigated, but they suggest that discrete subcortical neural populations are able to influence anti-tumor immunity via the sympathetic nervous system. In combination with findings involving HO neurons (discussed above), this links arousal circuitry to both anti-tumor immunity and systemic energy balance. 

Prior research suggests that chronic circadian disruption (e.g., via shift work, trans meridian flight) is associated with the development and progression of a variety of cancer types in both humans and rodent models [3,250,251,252]. In a proof-of-principle experiment, van Dycke and colleagues demonstrated that chronic circadian disruption (through repeated inversions of the light/dark cycle) accelerated spontaneous breast tumor development in a mouse model of breast cancer reflecting Li-Fraumeni syndrome [253]. Using cre-dependent p53 deletion, researchers were able to restrict primary cancer formation to mammary epithelial cells (*WAP-Cre::p53^fl/fl^)*. In this model, mice normally develop breast tumors spontaneously around 35 weeks of age. When exposed to the circadian disruption paradigm for many weeks, which significantly disrupts behavioral rhythms and sleep/wake dynamics, they developed tumors ~8 weeks sooner (~17% earlier) than littermates that were not exposed to the L/D inversion protocol. This study was the first to provide causal evidence linking light-induced circadian disruption and spontaneous tumor development in mice. Whether the tumors themselves further exacerbated sleep/circadian disruption remains to be determined. However, the use of a ‘human like’ transgenic model in this study is a significant step above the syngeneic models we discuss previously, which are sometimes described as an intermediate step between cell culture and cancer models (also known as ‘animal culture’) [254].

Building on these findings, Papagiannakopoulos and colleagues examined the influence of genetic and environmental circadian disruption on tumor development in a model of lung cancer [255]. The researchers used this model (*K-ras^LSL-G12D/+^*;p53^flox/*flox*^ *(KP)* mice) to see the effects of a jet-lag circadian disruption schedule on tumor growth, metabolism, and proliferation. Upon cre-mediated recombination, chronic jet-lag enhanced tumor growth, energy consumption, and proliferation. A nearly identical phenotype emerged when the mode of circadian disruption was via genetic deletion of core clock genes *Per2* and *Arntl1* in tumor cells (using *Kras^LA2/+^* mice to model spontaneous lung cancer development). Cells lacking these clock genes were highly proliferative in culture and more sensitive to transformation than cells with an intact clock. Additionally, *Per2* deficient cells drastically altered their energetic profiles and metabolic signature, secreting substantial amounts of the energy substrates glucose, lactate, and glutamine. When the authors examined human patient tumor samples, they observed significant reductions in the expression of nearly all core clock components (except for *clock*), suggesting that circadian disruption in cancer is conserved in humans. Using behavioral sleep strategies (e.g., cognitive behavioral therapy (CBT) for insomnia) or circadian treatment modalities (e.g., light therapy) may aid cancer elimination by enforcing rhythmic clock gene expression. 

As we discussed previously, cancer-induced changes in energy balance are enacted in order to sustain proliferative growth and meet metabolic demand [2]. Otto Warburg was the first to systematically describe how tumors drastically alter their energy production strategies (i.e., rely on glycolysis rather than oxidative phosphorylation; Warburg Effect) [256,257,258]. In many cancers, this results in the accumulation of inflammatory molecules and metabolic ‘waste’ from the tumors, which can influence systemic physiology. For example, cancer-induced elevations in circulating lactate can influence the activity of neurons involved in energy balance and food intake, including agouti-related protein (AgRP) neurons in the arcuate nucleus of the hypothalamus [259,260]. Tumor derived lactate influences food intake via its actions on the adenosine monophosphate kinase/methylmalonyl CoA (AMPK) signaling pathway within the hypothalamus, but it does not seem to be sole responsible for cancer-induced anorexia/cachexia [259]. How tumor-induced changes in circulating lactate influences the activity of arousal-related neural populations is completely undescribed and could lead to an understanding of the interplay between tumors, immunity, metabolism, and sleep disruption.

More recently, several studies have implicated calcitonin-gene related peptide (CGRP)-expressing neurons in the parabrachial nucleus (PBN) in general arousal, CO_2_ sensing, and cancer-associated cachexia/anorexia. Activation of these cells promotes rapid arousal from sleep, and they play a major role in the awakening effect of hypercapnia to putatively protect the sleeper from getting inadequate oxygen [261]. Using a mouse model of cancer cachexia/anorexia, Schwartz and colleagues investigated CGRP neural activity and its relation with food intake and metabolic state during tumor progression [262]. In anorexic mice harboring cancer, CGRP neurons were aberrantly and constitutively active, a phenotype that usually emerges after eating a large meal to signal meal termination [263]. This suggests that normal homeostatic mechanisms regulating food intake and energy balance become deregulated by cancer, driving debilitating side effects like anorexia/cachexia and fatigue. Inhibition of PBN^CGRP^ neurons using cre-dependent tetanus toxin normalized food intake in tumor-bearing mice, which was associated with improvements in downstream signaling pathway function in the oval subnucleus of the bed nucleus of the stria terminalis (ovBNST; also called the extended amygdala) and central amygdala (CeA). A similar rescue phenotype was observed when cellular inhibition was achieved using DREADDs (hM4Di), suggesting that the improvements were not due to destruction of these cells, but through their inhibition and downstream normalization of output. Further work is needed to examine pre-synaptic partners of these neurons (including the hunger-inducing AgRP neurons in the arcuate nucleus), and how they become deregulated in the context of cancer and/or cancer treatment.

## 11. Conclusions and Unanswered Questions

Advances in technology (e.g., calcium imaging, optogenetics) that allow for the manipulation and monitoring of neural circuitry has shed new light on how cancer-induced changes in physiology are communicated to the brain. Depending on the timing and valence of these inputs, distinct subcortical circuits (e.g., hypocretin/orexin) respond by altering their activity in an attempt to restore homeostasis. As a consequence, cancer-associated co-morbidities develop, including sleep/circadian disruption, systemic inflammation, metabolic reorganization, and anorexia/cachexia [1]. As many of these circuits reciprocally contribute to systemic physiology (e.g., VTA-DA neurons), understanding how these pathways operate in the context of cancer will undoubtedly lead to new therapeutic targets for cancer inhibition and elimination.

Essential questions regarding brain-cancer crosstalk still remain unanswered. Specifically, three broad areas need to be addressed: (1) What metabolites, cytokines, or other signals become deregulated in cancer and reach the brain?; (2) How do these (and neural) inputs influence the activity or connectivity of the brain; and (3) How do cancer-induced changes in neural dynamics contribute to changes in physiology and behavior? Beyond these, understanding the heterogeneity of tumor-brain communication with respect to cancer types and stages will need to be addressed in order to develop targeted and generalizable treatment strategies. 

The targeted stimulation of specific nuclei/subnuclei that become deregulated by cancer is a potential avenue for overcoming resistance to established anti-cancer therapies (e.g., immunotherapy). Data on how central neural stimulation influences peripheral physiology is sorely needed to understand how brain-centered therapies could augment anti-cancer immunity. As we discussed above, the enhancement of midbrain dopaminergic signaling (via Gq-coupled DREADDs) alters both innate and adaptive immunity, leading to tumor suppression via sympathetic modulation of myeloid-derived suppressor cells in the bone marrow [65,66]. Expanding this approach to other nuclei will allow us to construct a neuroimmune effector map that we can manipulate to enact specific changes in hematopoiesis and physiology critical for anti-tumor immunity. In humans, deep brain stimulation of the subthalamic nuclei safely and reversibly promotes sympathetic activation, with putative enhancements in immune responses [264]. Advancements in non-invasive neuromodulation techniques (e.g., ultrasound) will allow unobstructed access to immunologically-relevant circuits. Although beyond the scope of this review, we appreciate that a variety of hormones and reactive oxygen/nitrogen species may influence brain-tumor cross-talk. Further work should focus on these interactions in tandem with other physiological signals. Additionally, behavioral therapies that promote positive and rewarding experiences (e.g., engaging dopaminergic signaling, reducing stress) can be designed to facilitate cancer suppression [265,266]. 

Cancer-induced changes in energy balance offer an opportunity to modulate relevant neural circuits (e.g., AgRP, POMC, HO) to not only improve quality of life, but reduce energy availability to the tumor. As we discussed above, the inhibition of HO signaling rescued metabolic abnormalities and enhanced sleep in a mouse model of non-metastatic breast cancer [10]. Further, the inhibition of aberrant parabrachial nucleus CGRP neural activity greatly improved measures of anorexia/cachexia and fatigue in a mouse model of lung cancer [262]. Beyond direct neuromodulation, repurposing drugs that influence food intake, appetite, and energy balance (e.g., metformin) provides attractive approaches for adjuvant cancer therapy [267].

Cancer chronotherapy, which takes advantage of circadian rhythms in metabolism and detoxification, allows treatment to be administered at times that coincide with peak effectiveness and the lowest for potential side-effects [268,269]. Indeed, research has demonstrated that chronotherapy can significantly limit liver toxicity and inflammation in response to chemotherapeutics like cyclophosphamide and doxorubicin [253,270]. Efforts have turned to the development of novel clock enhancing molecules (CEMs) that can phase-advance, delay, or increase the amplitude of circadian rhythms. Nobiletin, a flavinoid found in citrus peel, acts to increase the amplitude of circadian oscillations in a dose-dependent manner [271]. As discussed previously, blunted circadian rhythms in physiology and behavior are strong predictors of mortality in cancer [4,6], suggesting that boosting circadian amplitude could promote survival. Indeed, nobiletin administration is sufficient to halt lung, breast, ovarian, and colorectal cancer progression in multiple mouse models [272,273,274,275], and patents have been issued for the use of nobiletin in the treatment of cancer [276]. Combining CEMs with chronotherapy offers a powerful approach to treat cancer with limited or negligible side-effects. Pursuing these avenues of research will help us to develop anti-cancer treatments and will also lead to basic discoveries relevant to brain-body cross-talk.

## Abbreviations

5HT	Serotonin
AKT	Protein kinase B
BBB	Blood brain barrier
Bcl-2	B-cell lymphoma 2
Bcl-xL	B-cell lymphoma-extra large
bFGF	Basic fibroblast growth factor
CCK	Cholecystokinin
CCL	C-C motif chemokine ligand
COX2	Cyclooxygenase-2
CRF	Corticotropin releasing factor
CRFR1	Corticotropin releasing factor receptor 1
DR	Dorsal raphe
EEG	Electroencephalogram
EGF	Epidermal growth factor
EMG	Electromyogram
FGF	Fibroblast growth factor
GABA	Gamma-Aminobutyric acid
GH	Growth Hormone
GHRH	Growth hormone-releasing hormone
GHSR	Growth hormone secretagogue receptor
GnRHR	Gonadotropin releasing hormone receptor
HcrtR1	Hypocretin receptor 1
HGF	Hepatocyte growth factor
HO	Hypocretin/Orexin
IGF	Insulin-like growth factor
IL-1	Interleukin-1
IL-10	Interleukin-10
IL-1R	Interleukin-1 receptor
IL-2	Interleukin-2
IL-4	Interleukin-4
IL-6	Interleukin-6
IL-8	Interleukin-8
K-ATP	ATP sensitive potassium channel
LC	Locus coeruleus
LepRb	Long-form leptin receptor
LH	Lateral Hypothalamus
LPS	Lipopolysaccharide
MAPK	Mitogen-activated protein kinases
MCH	Melanin concentrating hormone
MCHR1	Melanin concentrating hormone receptor 1
Mcl-1	Induced myeloid leukemia cell differentiation protein
mGluR	Metabotropic glutamate receptor
MMP	Matrix metalloproteinases
NE	Norepinephrine
NF-κB	Nuclear factor kappa-light-chain-enhancer of activated B cells
NO	Nitric oxide
NREM	Non-rapid eye movement
NREM	Non rapid eye movement
Ob-R	Leptin receptor
PDGF	Platelet-derived growth factor
PGE-2	Prostaglandin E2
PI-3K	Phosphoinositide 3-kinase
REM	Rapid eye movement
REM	Rapid eye movement
sIL-6R	Soluble interleukin-6 receptor
STAT3	Signal transducer and activator of transcription 3
TGF-b	Transforming growth factor beta
TNF-a	Tumor necrosis factor alpha
TNFR2	Tumor necrosis factor receptor 2
VEGF	Vascular endothelial growth factor
VLPO	Ventrolateral preoptic area
VTA	Ventral tegmental area
WASO	Wake after sleep onset
